# Data Integration to Support Child Care Business Sustainability: Iowa’s Child Care Connect

**DOI:** 10.23889/ijpds.v9i5.2880

**Published:** 2024-09-18

**Authors:** W. Todd Abraham, Heather Rouse, W. Todd Abraham

**Affiliations:** 1 Iowa State University

Working parents need access to childcare, and the unprecedented COVID-19 pandemic highlighted critical gaps in data system capacities to meet this need. This was particularly acute in the US where many child care providers closed their doors permanently because they could not sustain their business. The state of Iowa estimates losses over $930 million per year to businesses and individuals due to a lack of childcare. Childcare businesses are not sustainable if they cannot fill slots and be consistently paid for care – and families need to be able to find care when they need it to fill those slots. Iowa is addressing this gap by developing a real-time, integrated data solution that includes historically disparate data systems to better identify childcare supply and demand. Through intensive community engagement processes including leadership from state agencies, the development team created a system that includes multiple technology solutions for real-time data sharing; a secure cloud-based system for storage, integration, and analysis; and two public-facing applications that allow data to be aggregated and shared for strategic decision-making as well as used by families to identify available care when and where they need it. This presentation will discuss challenges in cross-sector data linkage with a focus on child care providers, families, and population demand. It will include an overview of the development process, governance structure, and analytic uses of the system. Highlights include identification of real-time vacancies by age group, geography, quality rating, and in relation to estimated potential demand using additional integrated data resources.

